# Novel Biomarkers in Lung Cancer and Chronic Lung Diseases: From the Systematic Perspective of *Yin–Yang* Balance

**DOI:** 10.3390/jcm11154275

**Published:** 2022-07-22

**Authors:** Mi-Kyung Jeong, Jaemoo Chun, In-Jae Oh

**Affiliations:** 1 KM Convergence Research Division, Korea Institute of Oriental Medicine, Daejeon 34054, Korea; oiny2000@kiom.re.kr (M.-K.J.); jchun@kiom.re.kr (J.C.); 2 Department of Internal Medicine, Chonnam National University Medical School, Hwasun 58128, Korea; 3 Lung and Esophageal Cancer Clinic, Chonnam National University Hwasun Hospital, Hwasun 58128, Korea

New approaches to personalized medicine are made possible by the discovery of biomarkers. Biomarkers are molecular characteristics of a disease that can be used to help make decisions about treatment. Through the recognition of novel biomarkers, it has become possible to identify subsets of patients who benefit from specific targeted therapies [1]. The success of targeted anticancer therapies and new immunotherapeutic approaches has created a new paradigm of personalized therapy and has also led to the accelerated development of new drugs for lung cancer treatment [2]. Additionally, several novel biomarker studies on early stage lung cancer have been published [3,4]. In addition, chronic lung diseases such as chronic obstructive pulmonary disease (COPD), interstitial lung disease (ILD), tuberculosis, and other smoking-related diseases are major causes of morbidity and mortality worldwide and result in economic and social burdens that are both substantial and increasing. To improve the prognosis of chronic lung disease, customized approaches based on novel biomarkers are being conducted, especially in the fields of COPD [5,6] and ILD [7,8].

Recently, cancer immunotherapy with immune checkpoint inhibitors (ICIs) has led to a major paradigm shift in the treatment of advanced-stage cancers. Unlike conventional therapeutic strategies that exert direct cytotoxic effects on tumor cells, ICIs, which are monoclonal antibodies targeting the immune-checkpoint proteins CTLA-4, PD-1, and PD-L1, block the inhibitory signals on T-cells and reinvigorate the anti-tumor immune response [9,10]. To date, ICIs have largely been used for their durable responses and survival benefits in patients with various type of cancers, including non-small lung cancer (NSCLC). However, its efficacy remains limited. The results of numerous clinical trials showed that only a small proportion of patients responded to ICIs in NSCLC. Additionally, there are several problems, such as acquired resistance, various immune-related adverse events, and hyper-progression in some patients receiving ICI therapy. Due to the dichotomous clinical outcome of ICI therapy, a great deal of effort has been carried out to identify the biomarkers for predicting which patients respond to ICI therapy. However, immune response cannot be fully understood with changes related to the proliferation, exhaustion, and differentiation of one or two immune parameters due to the complex and ‘multifactorial nature of cancer immune interactions’ [11]. A broader perspective of the immune system and the dynamic changes of immune factors is thus required.

Pattern identification, also known as syndrome differentiation, is an essential diagnostic system in traditional East Asian medicine and is used to examine imbalances in the entire body system. It is defined as comprehensively analyzing the clinical symptoms and signs to provide personalized treatment for each patient [12]. According to traditional medicine theory, from a systematic perspective, diseases occur due to a decline in immunity, which is maintained by a balance between *Yin* and *Yang*. *Yin* and *Yang* represents two interrelated components of a duality which integrate as a whole with dynamic interactions, such as mutual confrontation and promotion. This concept also exists as ‘homeostasis’ in modern medicine, which proposes a balance in the entire body system for maintaining a healthy state, from external or internal pathological factors. Disease treatment with traditional medicine aims to restore balance in immune status with the harmonization of *Yin* and *Yang* [13]. In this regard, increasing evidence suggests that the balance between the immunostimulatory and immunosuppressive factors in the tumor microenvironment (TME) could determine the ICI response and prognosis of patients with cancer. For instance, increased ratios of CD8 to regulatory T (Treg) cells or effector T cells to Treg cells in the TME are correlated with good clinical outcomes in cancer patients [14]. The results of many studies have shown that tumor-infiltrating CD8 T cells, Th1 cells, and type 1 macrophages were associated with prolonged survival; in contrast, immune-suppressive cells such as Treg cells, type 2 macrophages, and myeloid-derived suppressor cells were associated with poor outcome [14,15,16]. Moreover, Th1/Th2 balance is critical in the immune response to cancer. A large number of cytokines produced by Th1 or Th2 cells regulate various immune signaling processes and interactions between target antigen and immune cells. Th1/Th2-mediated immunity has bidirectional properties that inhibit immune response by shifting from Th1 to Th2 balance, or vice versa activate it by shifting from Th2 to Th1 balance in cancer [17,18]. This perspective of balance regarding immune status could be interpreted as the *Yin* and *Yang* balance in traditional medicine. Excessive *Yin* suggests an immune suppressive state in tumor immunity, with a predominance of immune regulation processes, such as increased Th2-mediated immune response, decreased tumor antigenicity, and increased immune-checkpoint proteins. In contrast, excessive *Yang* represents an immune activation state, with a predominance of inflammatory process, such as increased Th1-mediated immune response, which could consequently lead to an auto-immune reaction [19]. The cold–heat pattern identification, representing the principle of *Yin* and *Yang*, is used as a basic diagnosis in clinical fields. Significant differences have been reported for immune status and treatment response based on the cold–heat patterns in patients with NSCLC treated with ICIs [20].

Although there is still a lack of clinical evidence for pattern identification in cancer research, a systematic perspective based on the *Yin* and *Yang* principles for pursuing balance in immune status could provide a new possibility for comprehensively understanding the dynamic changes in the immune system. Hopefully, further integrative research with pattern identification will provide insight in biomarkers for personalized medicine.
